# Tumor endothelial cell-derived cadherin-2 promotes angiogenesis and has prognostic significance for lung adenocarcinoma

**DOI:** 10.1186/s12943-019-0987-1

**Published:** 2019-03-04

**Authors:** Huiqin Zhuo, Yan Zhao, Xiao Cheng, Mao Xu, Lin Wang, Lingyun Lin, Zhi Lyu, Xuehui Hong, Jianchun Cai

**Affiliations:** 1 Department of Gastrointestinal Surgery, The Affiliated Zhongshan Hospital, Xiamen University, Xiamen, 361004 Fujian China; 2 Institute of Gastrointestinal Oncology, School of Medicine, Xiamen University, Xiamen, 361004 Fujian China; 3 Central Laboratory, The First Hospital Affiliated to Xiamen University, Xiamen University, Xiamen, 361004 Fujian China; 4 Respiratory Department, The Affiliated Zhongshan Hospital, Xiamen University, Xiamen, 361004 Fujian China

**Keywords:** Tumor-derived endothelial cell, EC proteome profile, CDH2 expression, Non-small cell lung carcinoma, Heterogeneity

## Abstract

In lung cancer, antiangiogenic strategies targeting tumor-derived endothelial cells (TECs) afford a survival advantage, but the characteristics of TECs have not been comprehensively elucidated. Herein, high-purity (> 98%) TECs were obtained, and these cells retained expression of EC markers and exhibited high viability. ITRAQ-2DLC-MS/MS was performed to profile the proteome and the heterogeneity of ECs. Only 31 of 1820 identified proteins were differentially expressed between adenocarcinoma (ADC)- and squamous cell carcinoma (SCC)-derived TECs (TEC-A and TEC-S, respectively), and cadherin-2 (CDH2) was the most significantly upregulated protein in TEC-A samples. Positive immunostaining for CDH2 (score > 3) was significantly more frequent in the endothelium of ADC tissues than in that of SCC tissues. Loss- or gain-of-function analysis showed that CDH2 significantly promoted in vitro and in vivo angiogenesis and sensitivity to the antagonist exherin. The MAPK/ERK and MAPK/JNK signaling pathways may play crucial roles in CDH2-induced HIF-1α/VEGF-mediated angiogenesis. Moreover, high CDH2 expression in TECs was significantly associated with tumor stage, visceral pleural metastasis, and decreased overall survival in patients with ADC but not SCC. Together, these data indicate the importance of CDH2 in angiogenesis and highlight its potential both for antiangiogenic therapy and as a candidate prognostic marker for ADC.

**Electronic supplementary material:**

The online version of this article (10.1186/s12943-019-0987-1) contains supplementary material, which is available to authorized users.

Lung cancer (LC) has long been considered the leading cause of cancer-related deaths worldwide [1]. Many new antiangiogenic strategies (mainly focusing on tumor-derived endothelial cells (TECs)) have been developed, resulting in improved progression-free survival [2]. Therefore, it is necessary to comprehensively elucidate the characteristics of TECs to improve the success of antiangiogenic strategies for LC. Non-small cell lung carcinoma (NSCLC) mostly comprises squamous cell carcinoma (SCC, ~ 28%) and adenocarcinoma (ADC, ~ 48%), which have distinct clinical characteristics, histological presentations, proteomic profiles, metabolic phenotypes, immune cell signatures and gene expression subtypes [3–5]. For example, ADC commonly expresses the type II pneumocyte markers SP-C and TIF1; in contrast, SCC preferentially expresses the basal cell markers Trp63 and Krt5/14 [4]. In addition, the expression of glucose transporter GLUT1, is significantly elevated in SCC [5]. The clinical efficacy of currently approved antiangiogenic therapies (i.e., the anti-VEGF inhibitor bevacizumab and the anti-VEGF receptor 2 [VEGFR2] inhibitor ramucirumab) differs between SCC and ADC [6]. However, only a few strategies have been employed to focus on LC-derived TECs because of analytical limitations [7, 8], and reports on the heterogeneity of TECs are rare. To our knowledge, large-scale proteomics analysis of ECs in lung ADC, ADC-related TEC-selective proteins, or the heterogeneity of TECs between the ADC and SCC subtypes has not yet been reported. Therefore, we focused on the mechanism of the most important proteins responsible for heterogeneity to facilitate the development of potential therapeutic strategies.

## Results/discussion

### EC protein expression profiling based on iTRAQ analysis

**Fig. 1:**
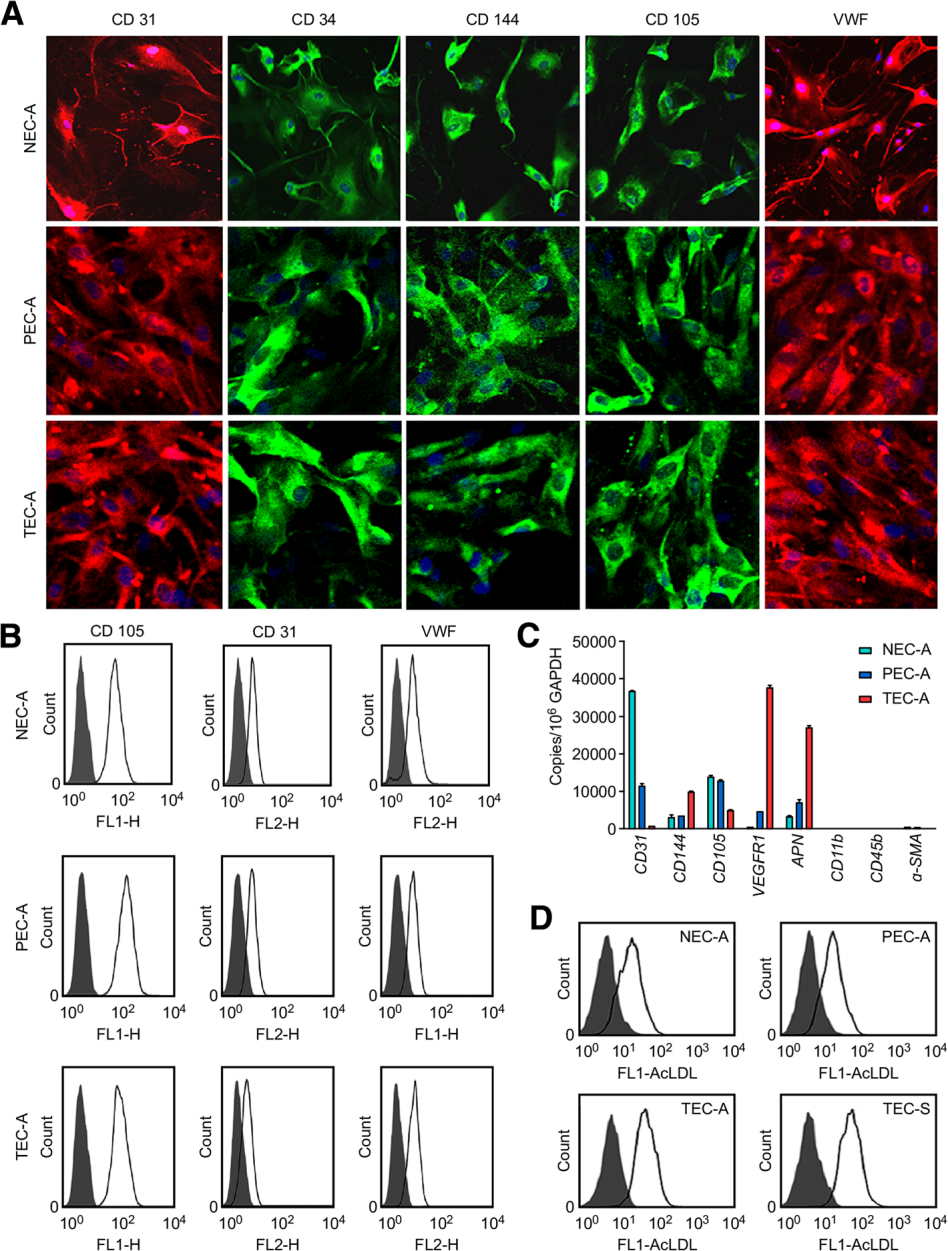
Assaying the expression of endothelial markers and AcLDL uptake in normal tissue-, paratumor- and tumor-derived ECs. **a** Positive immunoreactivity with antibodies specific for CD31 (red), CD34 (green), CD144 (green), CD105 (green), and VWF (red) using NEC-A (normal), PEC-A (paratumor), and TEC-A (tumor) tissues from six patients with lung ADC. Nuclei were stained with DAPI (blue). **b** Expression of the EC markers CD31 and VWF and the microvascular EC marker CD105 in NEC-A, PEC-A, and TEC-A tissues was evaluated by flow cytometry. **c** The mRNA expression levels of *CD105*, *CD31*, *CD144*, *VEGFR1*, *APN*, CD11b, CD45b, and α-SMA in NEC-A, PEC-A, and TEC-A tissues. The data were normalized to *GAPDH* expression and shown as the fold change compared to NECs. **d** Alexa Fluor® 488 AcLDL uptake assays. Uptake efficiency in NEC-A, PEC-A, TEC-A, and TEC-S (from tumor tissues of six lung SCC patients) was detected by flow cytometry

Primary ECs that were positive for expression of EC markers (CD31, CD34, CD144, CD105, and VWF) but negative for expression of a vascular smooth muscle cell (VSMC) marker (*α-SMA*) and monocyte markers (*CD11b* and *CD45b*), with high purity and viability (CD105 expression > 98%, AcLDL uptake > 90%), were prepared (Fig. 1; Additional file 1). A total of 1820 proteins were identified. Gene set enrichment analysis of the differentially expressed proteins among ADC-related ECs shown in Additional file 2: Figure S1, including the 81 proteins unique to TECs from ADC (TEC-A) (compared to paratumor-derived ECs from ADC (PEC-A) and normal tissue-derived ECs (NEC-A)), which were mainly involved in transcription activity, protein prepare, hydrogen peroxide catabolic processes, NF-κB transcription factor activity.

**Fig. 2:**
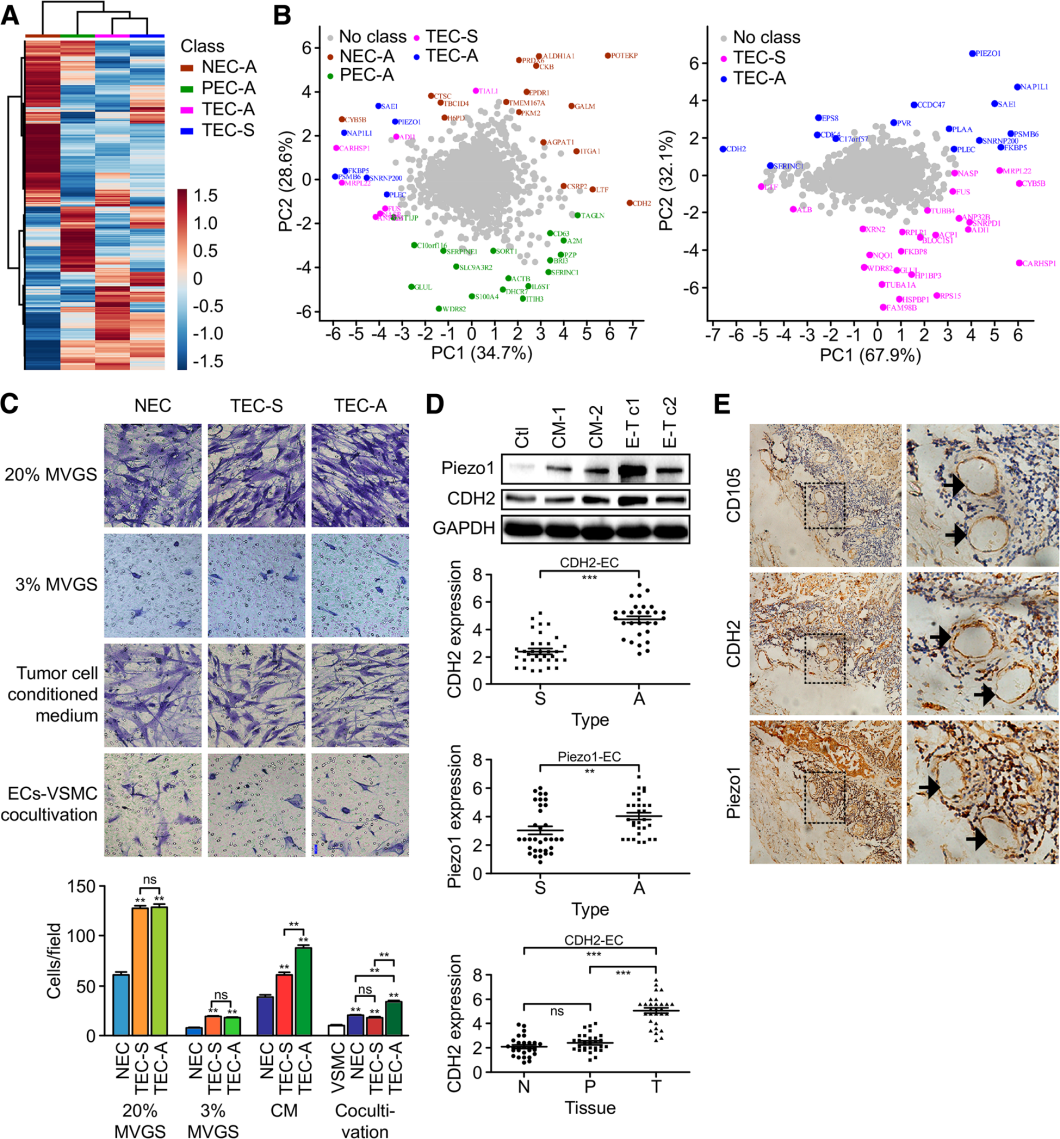
Protein expression patterns and heterogeneity of CDH2 and Piezo1 expression in ADC- and SCC-derived ECs. **a** Cluster map comparing the protein expression patterns of NEC-A, PEC-A, TEC-A, and TEC-S. Functional category gene enrichment tests were performed using heatmap.2 and the gregmisc package in the R statistical environment (http://cran.r-project.org/web/packages/gplots). Red indicates higher expression levels, and blue indicates lower expression levels in the two cell types. **b** The first and second principle components, PC1 and PC2, from the principle component analysis (PCA). The complete proteomic data sets for the four EC groups were subjected to PCA analysis to capture the maximum variance among ECs, using the SIMCA-P + V12.0.1 software package. The principal components (proteins from the original four groups) were marked with the corresponding name and group color. **c** Migration assay. The migration of NEC (pool of NEC-A and NEC-S), TEC-S, and TEC-A cells (1.8 × 10^4^) was detected using transwell chambers (6.5 mm, 8.0-μm), after induction by M131 medium supplemented with 20% or 3% MVGS or tumor cell CM at 37 °C for 12 h. To study EC-induced VSMC migration, cocultivation was established by plating VSMCs (2.4 × 10^4^) onto the filter inserts and transferring to the EC monolayer (5.0 × 10^6^), then incubating in M131 medium supplemented with 0.2% fetal bovine serum at 37 °C for 16 h. The cells were stained with 20% Giemsa solution. The number of migrated cells was counted in five randomly selected fields under a microscope (200×). **d** Heterogeneity of CDH2 and Piezo1 expression in ADC- and SCC-derived ECs. Expression of Piezo1 or CDH2 in HUVECs after being cultured in CM-1, CM-2 (SPC-A-1 or L-78 cell-conditioned medium, respectively), Coculture 1 (cocultured with the ADC cell line SPC-A-1), or Coculture 2 (cocultured with the SCC cell line L-78). **e** Consecutive sections were prepared to detect CD105, CDH2, and Piezo1 expression in the endothelial cells of tumor samples from 68 patients (36 with SCC and 32 with ADC), and CD105 expression was detected as a positive control. The fields shown in dotted lines were enlarged two-fold in the right column, and microvessels are identified using black arrows, based on positive staining for CD105. Expression of Piezo1 or CDH2 in ADC- and SCC-derived ECs, and CDH2 in ECs from tumor tissues (T) and paired paratumor (P) and normal (N) tissues of 32 patients with ADC were statistically analyzed. The intensity of staining (IS) was scored as 0: negative, 1: weak, 2: moderate and 3: strong. The percentage of positive (PP) cells was scored as 0 (PP ≤ 5%), 1 (6% ≤ PP ≤ 25%), 2 (26% ≤ PP ≤ 50%), 3 (51% ≤ PP ≤ 75%), and 4 (PP ≥ 75%). The staining in endothelial cells was scored by multiplying the PP by the IS (immunoreactive score = PP × IS): 0 (score: 0–2, negative), 1+ (score: 3–4, moderately positive), 2++ (score: 5–6, strongly positive), and 3+++ (score: 7–8, very strongly positive)

The proteomic profiles were similar in TECs by heatmap analysis (Fig. 2a). A few principal components accounted for 63.3% (PC1 and PC2) of the total data variation, and the locations of the TECs were very close together. Then, the TEC proteomic profiles were analyzed in pairs (Fig. 2b). The results show the proteins (including CDH2, Piezo1, EPS8, NAP1L1, FAM98B, HSPBP1, RPS15 and so on) responsible for segregation into two groups. Only 31 proteins (see Additional file 3: Table S1) were differentially expressed in both groups, among which CDH2 and Piezo1 were the two most significantly upregulated proteins in the TEC-A group. The expression levels of 15 significantly altered proteins were verified by western blotting (Additional file 4: Figure S2A). Similar changes were observed in PEC-A, TEC-A and tumor cell–EC cocultivation samples. Therefore, all types of isolated ECs provide a good basis for studying tumor angiogenesis and heterogeneity.

### Heterogeneity of lung cancer-derived ECs

EC migration and positive regulation of smooth muscle cells are two important biological processes relevant to angiogenesis. The migration of TECs induced by either M131 medium containing 20% or 3% MVGS or tumor cell-conditioned medium (CM) was significantly faster than that of NECs (pool of NEC-A and NEC-S), but TEC-A migration was even faster (*P* < 0.01) than that of TEC-S in the CM group. Moreover, during EC- VSMC cocultivation, although all types of ECs enhanced the migration of VSMCs, TEC-A did so most markedly (Fig. 2c).

The expression levels of Piezo1 and CDH2 were upregulated in primary human umbilical vein endothelial cells (HUVECs) cocultured with the lung ADC cell line SPC-A-1 but not the SCC cell line L-78 or CM from SPC-A-1 or L-78 cells (Fig. 2d). Positive immunostaining of Piezo1 and CDH2 (score > 3) was significantly more frequent in TEC-A than in TEC-S samples (*P* = 0.01 and *P* < 0.0001, respectively; Fig. 2e). In SCC, the expression of CDH2 was much higher in TECs than in NECs or PECs. Furthermore, the prognostic values revealed that high tumor CDH2 expression was related to a shorter survival time in LC patients (*P* = 0.017); this correlation was more significant in ADC (*n* = 720, *P* = 0.008) but not significant in SCC (*n* = 524, *P* = 0.220). However, no similar correlation between Piezo1 expression and survival time was observed (Additional file 4: Figure S2B). These data indicated that CDH2 likely plays more important functional roles in ADC development than in SCC development.

### Effect of CDH2 on angiogenesis

Loss- or gain-of-function analysis revealed that CDH2 expression significantly promoted EC proliferation, motility and capillary-like tube formation, whereas silencing endogenous CDH2 had the opposite effect (Additional file 5: Figure S3). A markedly increased density of neovessels (VWF-positive) was observed in Matrigel plugs containing CDH2-overexpressing ECs (Additional file 6: Figure S4A). Preclinical and clinical data have shown that a peptidic CDH2 antagonist (exherin, ADH-1) causes rapid tumor vascular disruption and apoptosis [9]. To date, limited research has been conducted on CDH2-expressing ECs. When treated with 0.4 mg/mL ADH-1 for 24 h, the CDH2 overexpression group (mainly located in the cell membrane) was significantly inhibited, but this effect was not obvious in the control group. ADH-1 induced apoptosis in a dose- and CDH2-dependent manner in ECs (Additional file 6: Figure S4B&C).

### Mechanism of CDH2-mediated angiogenesis

With CDH2 overexpression, epithelial cell markers (E-cadherin, EpCAM, and P-cadherin), neural and basal cell adhesion molecules (NCAM-1, NrCAM, and BCAM), leukocyte rolling and attachment molecules (L-, E-, and P-selectin), and strictly EC-specific adhesion molecules (VE-cadherin) were all downregulated. Moreover, proteins known to positively regulate leukocyte migration (RANTES, ENA-78, IL-6, MCP-2, and IP-1α) were also significantly downregulated. Three proteins involved in the VEGFR signaling pathway (VEGFA, VEGFD, and VEGFR3) were significantly upregulated. Furthermore, the phosphorylation levels of two major MAPK signaling pathways, namely, the extracellular signal-regulated kinase (ERK) and c-Jun N-terminal kinase (JNK) pathways, were significantly increased, and CDH2 was confirmed to induce HIF-1α, VEGF, and VEGFR3 accumulation (Additional file 7: Figure S5). Therefore, the MAPK/ERK and MAPK/JNK signaling pathways are likely to play crucial roles in CDH2-induced HIF-1α/VEGF-mediated angiogenesis.

### Clinical value of CDH2 in ADC

**Fig. 3:**
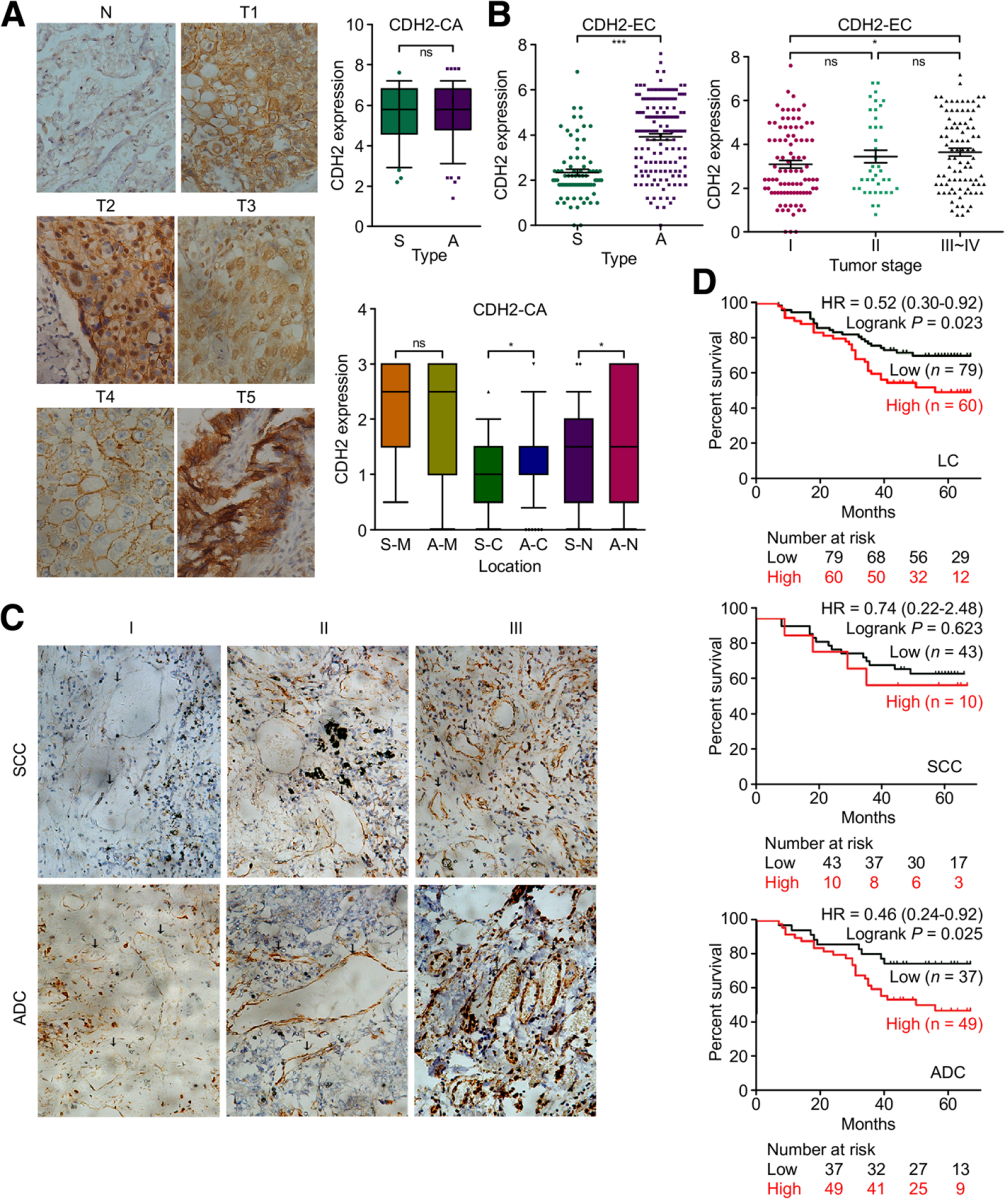
Aberrantly increased CDH2 protein expression in ADC-derived ECs by IHC analysis in 218 tumor tissues from lung cancer patients (141 with ADC and 77 with SCC). **a** IHC images of heterogenous CDH2 localization and CDH2 expression scores for cancer cells from SCC and ADC samples. IHC staining for SCC and ADC cancer cells was scored by PP × IS. CDH2 expression localization in cancer cells was further rescored by the intensity of staining (IS, score: 0~3) in membrane, cytoplasm and nucleus. **b**, **c** High CDH2 expression in TECs from ADC tissues. The significantly positive relationship between CDH2 expression and tumor stage, as well as typical IHC images displaying increased CDH2 expression during tumor progression are shown. Microvessels are indicated with black arrow, based on positive staining for CD105. **d** The overall survival of patients with lung cancer and differential CDH2 expression in TECs. The prognostic value of CDH2 expression was investigated in 139 of 218 patients with lung cancer with respect to survival information

The adjacent normal tissues were nearly negative for CDH2 expression, but tumor cells in the lung tissues exhibited very strong positive staining (Fig. 3a, clinical data were listed in Additional file 8: Table S2). The expression of CDH2 in cancer cells from SCC and ADC did not differ significantly (scored by PP × IS), but its localization in cancer cells was heterogeneous. Therefore, expression was further rescored by the intensity of staining (IS, score: 0~3) in the membrane, cytoplasm and nucleus. CDH2 expression in the cytoplasm or nucleus was significantly higher in cancer cells form ADC than in those from SCC (*P* < 0.05). Heterogeneity of CDH2 cleavage resulted in different regulatory behaviors related to cancer cell–cell adhesion and cell migration/ proliferation [10].

Positive CDH2 staining (score > 3) was seen in 22 and 66% of TECs from SCC and ADC tumors, respectively (Fig. 3b). High CDH2 expression in TECs was significantly positively related to ADC (*P* < 0.001) and tumor stage (*P* < 0.05) in LC patients, and positively related to tumor stage and visceral pleural metastasis in the ADC subtype (*P* < 0.05, Fig. 3b and c; Additional file 9: Table S3). Significantly poorer prognosis and overall survival were associated with patients with high TEC CDH2 expression and LC (*P* = 0.023) or ADC (*n* = 86, *P* = 0.025) but not SCC (*n* = 53, *P* = 0.623) (Fig. 3d). These data suggest that CDH2 may serve as a prognostic indicator of relative risk for patients with ADC.

## Conclusion

The proteomic profile of ADC-derived ECs was elucidated, and CDH2, which was uniquely upregulated in TEC-A, was proved to promote in vitro and in vivo angiogenesis and sensitivity to the antagonist ADH-1. The MAPK/ERK and MAPK/JNK signaling pathways may play crucial roles in CDH2-induced HIF-1α/VEGF-mediated angiogenesis. Strong CDH2 expression was significantly more frequent in TECs, and this expression pattern was associated with tumor stage, visceral pleural metastasis, and decreased overall survival in patients with ADC but not SCC. These data indicated the important role of CDH2 in angiogenesis, as well as its potential as both a new molecular target in combination with currently approved anti-angiogenic strategies and a candidate prognostic marker for ADC.

## Additional files

Additional file 1: Materials/Methods. (DOC 46 kb)

Additional file 2: **Figure S1.** Functional categorization based on Gene Ontology (GO) and KEGG pathway analysis. Functional categorization of the proteins that were (A) differentially expressed in both the TEC-A and PEC-A groups compared to the NEC-A group, (B) unique TEC-A proteins (compared to the NEC-A and PEC-A groups), and (C) unique PEC-A proteins (compared to the NEC-A and TEC-A groups). Only the top 10 biological processes and KEGG pathways are listed, with statistical significance assigned based on the corrected *P* < 0.05. (TIF 10537 kb)

Additional file 3: **Table S1** Proteins differentially expressed in lung adenocarcinoma-derived endothelial cells (TEC-A) in comparison to squamous cell carcinoma-derived endothelial cells (TEC-S). (DOC 68 kb)

Additional file 4: **Figure S2.** Verification of differential protein expression and the prognostic value of CDH2 and Piezo1 in patients with LC, using the Kaplan–Meier plotter database (http://kmplot.com/analysis/index.php?p=service&cancer= lung). Lysates from C-A (NEC-A), P-A (PEC-A), T-A (TEC-A), Pc-A (PEC-A cocultured with LTEP-α-2), and Tc-A (TEC-A cocultured with LTEP-α-2) cells were prepared from six additional lung ADC patients. Lysates from C-S (NEC-S), T-S (TEC-S), and TC-S (TEC-S cocultured with SK-MES-1) cells were prepared from the six lung SCC patients. The experiments were repeated at least in triplicate. (TIF 6384 kb)

Additional file 5: **Figure S3.** In vitro assays of CDH2 function in microvascular endothelial cell (MVEC), Ealy926, and HUVEC lines by overexpressing or knocking down CDH2 expression. (A) Efficient CDH2 overexpression or knockdown was confirmed by western blotting. (B, C) Cell survival and apoptosis assays of MEVCs and Ealy926 cells after CDH2 overexpression or knock down. (D, E) Cell migration and in vitro angiogenic activity of MVECs, Ealy926 cells and HUVECs following CDH2 overexpression or knock down. Cell numbers were counted in five randomly selected fields under a microscope. (TIF 13045 kb)

Additional file 6: **Figure S4.** Matrigel plug assay and ADH-1-induced apoptosis in CDH2-expressing cells. (A) Matrigel plug assay. Growth factor-reduced Matrigel matrix (0.25 Ml) supplemented with 8.0 × 10^5^ MVECs or Ealy926 cells (mock cells or clones stably expressing CDH2) was injected subcutaneously at the abdominal midline of six-week-old BALB/c nude male mice. Four mice were included in each group. After 10 days, the Matrigel plugs were split and sectioned for anti-VWF (red) and DAPI (blue) staining. The results from two randomly selected models are shown. (B, C) ADH-1-induced apoptosis in CDH2-expressing cells, as determined by in vitro assays. ADH-1 (0, 0.1, 0.2, or 0.4 mg/mL) was incubated with control and CDH2-overexpressing ED-25 cells for 24 h (confocal microscopy-based observations) or 48 h (FCA). Cells incubated with anti-CDH2 (green) and VE-cadherin antibodies (red) were observed with a confocal microscope. Apoptosis was detected using the Annexin V-FITC/PI Kit. (TIF 10193 kb)

Additional file 7: **Figure S5.** Mechanism of CDH2-mediated angiogenesis. Angiogenesis antibody (A) and molecular adhesion array (B). HUVECs were infected with an adenoviral vector directing the expression of CDH2 or a mock vector. Subsequently, the HUVECs were harvested and analyzed for protein expression using RayBiotech Human Angiogenesis Antibody Array C Series 1000 and Adhesion Molecule Array Q1. The blots were scanned with an InnoScan 300 Microarray Scanner and analyzed using ImageJ software. (C) CDH2 induced upregulation of VEGFA, VEGFR3, MMP-1, and HIF-1α in MVECs and ED-25 cells, as determined by western blotting. Activation of two major MAPK signaling pathways, namely, extracellular signal-regulated kinase (ERK) and c-Jun N-terminal kinase (JNK), was detected. The levels of phosphorylated ERK1/2, JNK, and c-Jun were detected in MVECs and ED-25 cells overexpressing CDH2. (TIF 7116 kb)

Additional file 8: **Table S2.** Clinical data for 218 lung carcinoma specimens examined in this study. (DOC 274 kb)

Additional file 9: **Table S3.** Expression of CDH2 in tumor-derived endothelial cell and its correlation with clinicopathological features of patients with NSCLC. (DOC 82 kb)

## Abbreviations

AcLDL: Acetylated low-density lipoprotein
ADC: Adenocarcinoma
BP: Biological processes
CDH2: Cadherin-2
CM: Cell-conditioned medium
ECs: Endothelial cells
GO: Gene Ontology
LC: Lung cancer
MVGS: Microvascular growth supplement
NSCLC: Non-small cell lung carcinoma
PCA: Principal component analysis
SCC: Squamous cell carcinoma
TECs: Tumor endothelial cells:
VEGF: Vascular endothelial growth factor
