# Supplementary material for: Tumor endothelial cell-derived cadherin-2 promotes angiogenesis and has prognostic significance for lung adenocarcinoma
Source: Mol Cancer. 2019 Mar 4;18:34. doi: 10.1186/s12943-019-0987-1 (PMC6399986; doi:10.1186/s12943-019-0987-1)
Supplement: Supplementary file 1 — Materials/Methods. (DOC 46 kb) [file 12943_2019_987_MOESM1_ESM.doc]

**Materials/Methods**

***EC isolation, culture, and characteristics***

All tissues used in this study were requested following approval by the Institutional Review Board of the Tumor Tissue Bank of Zhongshan Hospital (Xiamen University, Fujian, China). Written informed consent was obtained from all patients, who had no chemotherapy or radiotherapy prior to the operation. The tissues were collected according to the standard procedures of the International Society for Biological and Environment Repositories. Individuals were enrolled and followed-up for over 5 years. Lung ADC-derived TEC-A (from the cancer loci), PEC-A (<3 cm distant from the cancer loci), and NEC-A (>5 cm distant from the cancer loci) cells were prepared as we described previously, including enzymatic digestion, differential adherence, and anti-CD105 antibody-coupled magnetic cell sorting.

Total RNA was extracted using TRIzol (Thermo, USA) to ensure the purity of the primary ECs. The mRNA levels of *CD105*, *CD144*, *CD31*, *APN*, *VEGFR1*, *CD45*, *CD11b*, and *α-SMA* were measured in ADC-derived ECs.

Immunofluorescence staining and flow cytometric analysis (FCA) were performed to verify the quality and characteristics of ECs. The cells were imaged and observed with a Zeiss LSM780 confocal microscope equipped with the Axio Observer Z1 platform (Carl Zeiss GmbH, Oberkochen, Germany). The following five antibodies were used: mouse anti-human CD31, CD105 (Biolegend, USA), and CD34 (eBioscience, USA); sheep polyclonal anti-human VWF; and rat anti-human CD144 (Abcam, USA). Alexa Fluor® 488- or 555-conjugated secondary antibodies were chosen. For FCA, the cells were incubated separately with isotype controls or anti-CD105, anti-CD31, or anti-VWF antibodies. The samples were analyzed on a Beckman Gallios Flow Cytometer (Beckman Coulter, Inc., USA) at least in triplicate. The three markers were detected by FCA after every purification experiment.

ECs were incubated with acetylated low-density lipoprotein (AcLDL) labeled with Alexa Fluor 488 (Molecular Probes, USA) at a final concentration of 15 μg/mL for 5 h at 37ºC. The positive rate was detected by FCA.

***ITRAQ-2DLC-MS/MS analysis***

NEC-A, PEC-A, and TEC-A specimens isolated from 6 ADC patients were prepared for iTRAQ-2DLC-MS/MS analysis, and these tissues were pooled at passage 1. TEC-S samples purified from 6 SCC patients were obtained in our previous study [Ref. 8]. Pooled samples were used to overcome variations in individual patients and to facilitate identification of the predominant heterogeneities. Cells from each group were lysed, and proteins were digested and labeled with iTRAQ reagents: 113 tag (NEC-A), 114 tag (PEC-A), 115 tag (TEC-A), and 116 tag (TEC-S), following the manufacturer’s instructions (AB Sciex, USA). Labeled peptides were subjected to strong cation exchange (SCX) chromatography on an AKTA Purifier 100 system (GE Healthcare, USA) equipped with a polysulfoethyl column (5-μm particle size and 200-Å pore size, 100 × 4.6 mm, PolyLC, Inc., USA). A Q-Exactive Hybrid Quadrupole-Orbitrap mass spectrometer (Thermo Fisher Scientific, USA) equipped with an EASY-Nlc 1000 liquid chromatography system was used for LC-MS/MS experiments. Protein searches were performed against the human protein sequence database (IPI v3.87, file ipi.HUMAN. v3.87.fasta.gz) using Proteome Discoverer software version 1.3 (Thermo) with the MASCTO^TM^ and/or the SEQUEST® search engine. The parameters for ITRAQ-2DLC-MS/MS analysis and protein searches were set according to the method described in our previous report [Ref. 8]. The parameters for quantification by Proteome Discoverer version 1.3 were as follows: fold-change threshold of two; a 20 ppm fragment ion tolerance for the most confident centroid peak; and only peptides unique for a given protein or protein group were considered for relative quantification. Experimental bias was corrected by the software via normalization to the protein median in each sample, and this factor was then applied to all quantification results. Relative quantification was based on the ratio of the reporter ions.

***Bioinformatic analysis***

Various bioinformatic analysis approaches were used to gain insight into the functional distribution of the identified proteins. First, all proteins were functionally categorized by performing Gene Ontology (GO) analysis, and assignments were made based on the Human Protein Reference Database (HPRD). Next, we performed a functional category gene-enrichment test by heatmap.2 and the gregmisc package in the R statistical environment. Third, principal component analysis (PCA) was conducted using the SIMCA-P + V12.0.1 software package (Umetrics, Sweden). Finally, the identified proteins were assigned to biological pathways using Kyoto Encyclopedia of Genes and Genomes (KEGG) pathways and KEGG Environmental Information Processing (EIPP).

***Cell cocultivation and migration***

Human lung SCC cell lines (SK-MES-1 and L-78) and ADC cell lines (SPC-A-1 and LTEP-α-2) purchased from the Institute of Cell Biology (Shanghai, China) were used for tumor cell–EC cocultures. For the indirect coculture groups, transwell inserts (0.4-μm; Corning, USA) were used to separate the cancer cells (upper chambers) from the ECs (lower chambers). The ECs were harvested after a 48-h incubation.

The medium of SK-MES-1 and SPC-A-1 cells following culture in RPMI 1640 basic medium for 24 h was collected and filtered through a 0.22-μm membrane to acquire the tumor cell-conditioned medium (CM). M131 medium supplemented with 20% or 3% microvascular growth supplement (MVGS) or CM was placed in the lower compartment of the transwell (8.0-μm), and the ECs (1.8 × 10^4^) were prepared on the inserts. Then, the chambers were incubated at 37°C for 12 h.

For EC-induced vascular smooth muscle cell (T/G HA-VSMC) migration, VSMCs (2.4 × 10^4^) were plated onto the filter inserts. The filters were then transferred to an EC monolayer (5.0 × 10^6^) in M131 containing 0.2% fetal bovine serum (FBS, HyClone, USA)) and incubated at 37°C for 16 h. The filters were fixed with methanol and stained with 20% Giemsa solution. Transmigration was observed under the microscope, and the number of cells was counted in five randomly selected fields (200×).

**In vitro *assays of EC functions***

EC lines (Ealy926 and MVEC) from the Institute of Cell Biology were cultured in DMEM-F12 supplemented with 10% FBS. Full-length cDNA encoding human *CDH2* was obtained by PCR, cloned into the pLVX-IRES-NEO vector, and verified by DNA sequencing. *CDH2* expression was transiently suppressed using siRNA obtained from Sigma. The *CDH2* siRNA target sequences were 5′-CAGUCAACUGCAACCGUGUdTdT-3′ (sense) and 5′-ACACGGUUGCAGUUGACUGdTdT-3′ (antisense). Plasmids (pLVX-*CDH2* or control vector) or siRNAs (siRNA targeting *CDH2* or a nontargeting siCtrl siRNA) were transfected for 36 h into EC cells using Lipofectamine 2000 and Lipofectamine^TM^ RNAi MAX (Thermo, USA), respectively. Stably transfected clones were infected with adenoviral vectors and established by G418 selection. Protein expression was monitored by western blotting to ensure efficient transfection. Then, cell proliferation, migration, and apoptosis in ECs was detected in the CDH2 overexpression and knockdown groups and the corresponding control groups.

Briefly, 200 μL of Matrigel (BD Bioscience) was added to the wells of a 48-well culture plate and allowed to polymerize for at least 2 h at 37ºC. ECs (2 × 10^4^) were resuspended with serum-free medium and added to the prepared Matrigel. The cells were allowed to form polygonal structures for 6 h at 37ºC and imaged by light microscopy. Vascular branch crossing was counted in five nonoverlapping microscopic fields under 40× magnification for each condition.

***Matrigel plug assay***

Growth factor-reduced Matrigel (0.25 mL) containing MVECs or Ealy926 mock cells and a clonal cell line stably expressing CDH2 (8.0 × 10^5^ cells) was subcutaneously injected at the abdominal midline of six-week-old BALB/c nude male mice. Four mice were included in each group. After 10 days, the Matrigel plugs were split and frozen in OCT. The frozen sections were fixed with a 4% paraformaldehyde solution and prepared for staining with an anti-VWF antibody (for ECs) and nuclear staining with DAPI.

***Exherin-induced apoptosis in CDH2-expressing cells using* in vitro *assays***

Exherin trifluoroacetate (ADH-1, catalog number HY-13541A, MCE Corporation, USA), a CDH2 antagonist, was used to induce apoptosis. Exherin (0, 0.1, 0.2, 0.3, 0.4, 0.5, or 1.0 mg/mL) was incubated with control and CDH2-overexpressing cells (ED-25 or MVEC) for 24 or 48 h. Cells incubated with anti-CDH2 and VE-cadherin antibodies (Cell Signaling Technology Inc., CST, USA) were observed with a confocal microscope. Apoptosis was detected.

***Angiogenesis-related antibody and adhesion molecular arrays***

HUVECs infected with adenoviral vectors (CDH2 expression or the mock vector) were analyzed for protein expression using RayBiotech Human Angiogenesis Antibody Array C Series 1000 and Adhesion Molecule Array Q1 (RayBiotech, catalog numbers QAH-ANG-1000 and QAH-CAM-1, respectively), following the manufacturer’s instructions. The blots were scanned with an InnoScan 300 Microarray Scanner and analyzed with ImageJ software (Innopsys Inc., France). Proteins were analyzed by western blotting using antibodies specific for VEGFA, VEGFR3 (Abcam, USA), MMP-1 (Santa Cruz Biotechnology, USA), HIF-1α, phospho-ERK1/2, JNK, and C-Jun (CST).

***Immunohistochemical (IHC) staining***

Lung carcinoma specimens from patients with clinical data who underwent long-term follow-up were purchased for IHC analysis. Consecutive sections (4 μm) were used to detect CD105, CDH2, and Piezo1; CD105 was detected in clinical samples as a positive control. Rabbit monoclonal antibodies specific for human CD105 (1:200 dilution), CDH2 (1:200 dilution), and Piezo1 (1:250 dilution) were purchased from Abcam. Scoring of IHC data was performed by two certified pathologists at 40× magnification, and the scores were averaged. Consecutive images were obtained for determining CDH2 and Piezo1 staining, and vascularization was confirmed by CD105 staining. The intensity of staining (IS) was scored as 0: negative, 1: weak, 2: moderate and 3: strong. The percentage of positive (PP) cells was scored as 0 (PP ≤ 5%), 1 (6% ≤ PP ≤ 25%), 2 (26% ≤ PP ≤ 50%), 3 (51% ≤ PP ≤ 75%), and 4 (PP ≥ 75%). The staining in endothelial cells and cancer cells was scored by multiplying the PP by the IS (immunoreactive score = PP × IS): 0 (score: 0–2, negative), 1+ (score: 3–4, moderately positive), 2++ (score: 5–6, strongly positive), and 3+++ (score: 7–8, very strongly positive). Furthermore, the staining in the membrane, cytoplasm and nucleus of cancer cells was scored by the intensity of staining (IS, score: 0~3).

***Statistical analysis***

Statistical analysis was performed using SPSS Viewer 22 (SPSS, IL, USA) and GraphPad Prism 7. Qualitative variables were analyzed using the Kruskal–Wallis H test, *t*-tests, and the Mann–Whitney U test. A two-tailed *P-*value of ≤ 0.05 was considered statistically significant. Clinical data were analyzed with Pearson correlations and Partial correlation coefficients, which are used to exclude the effects of control variables. The log-rank (Mantel–Cox) and Gehan–Breslow–Wilcoxon tests were used for survival curve analysis.
