# Supplementary figures and images for: Tumor endothelial cell-derived cadherin-2 promotes angiogenesis and has prognostic significance for lung adenocarcinoma
Source: Mol Cancer. 2019 Mar 4;18:34. doi: 10.1186/s12943-019-0987-1 (PMC6399986; doi:10.1186/s12943-019-0987-1)

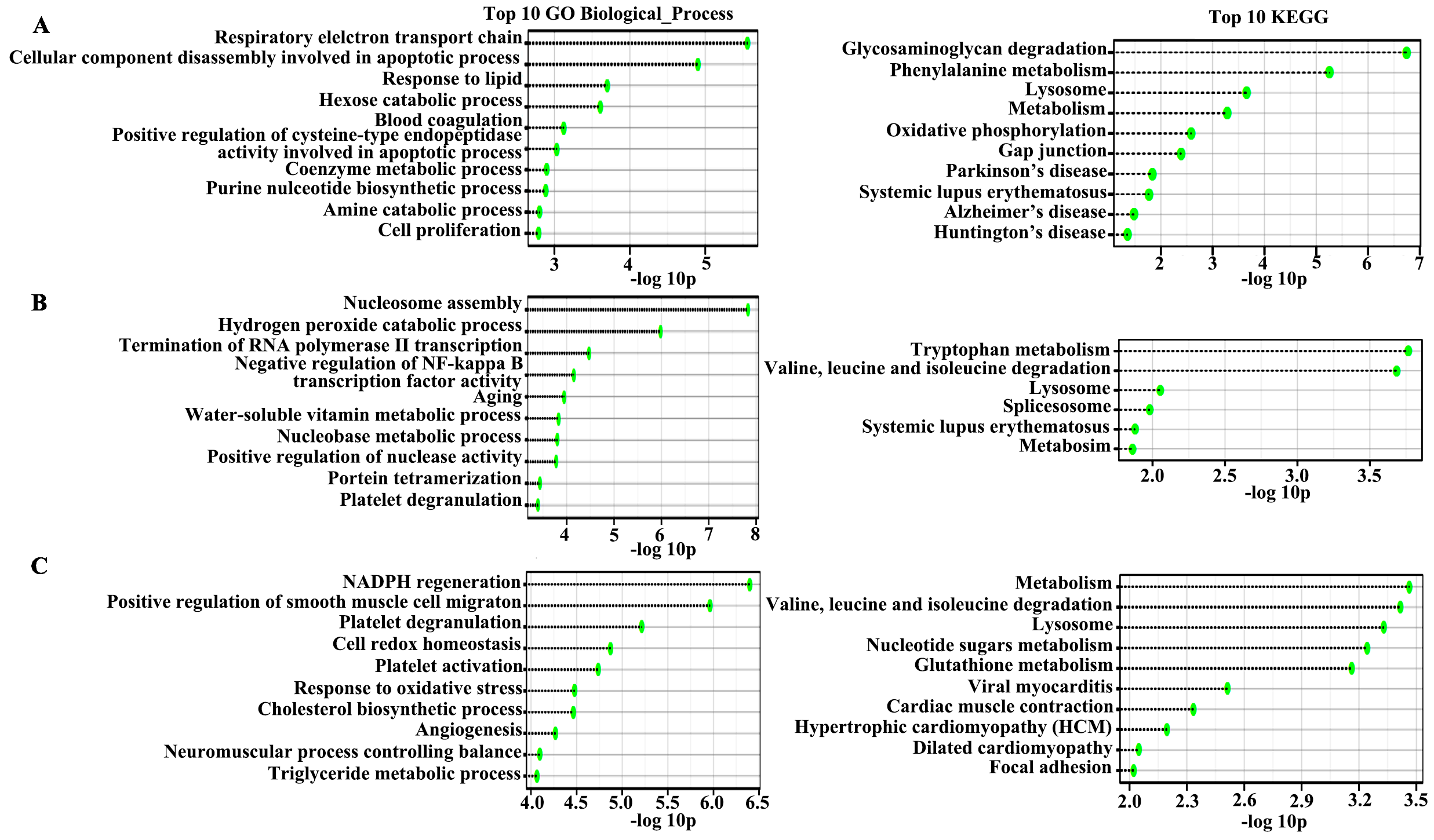

Supplement: Supplementary file 2 — Figure S1. Functional categorization based on Gene Ontology (GO) and KEGG pathway analysis. Functional categorization of the proteins that were (A) differentially expressed in both the TEC-A and PEC-A groups compared to the NEC-A group, (B) unique TEC-A proteins (compared to the NEC-A and PEC-A groups), and (C) unique PEC-A proteins (compared to the NEC-A and TEC-A groups). Only the top 10 biological processes and KEGG pathways are listed, with statistical significance assigned based on the corrected P < 0.05. (TIF 10537 kb) [file 12943_2019_987_MOESM2_ESM.tif]

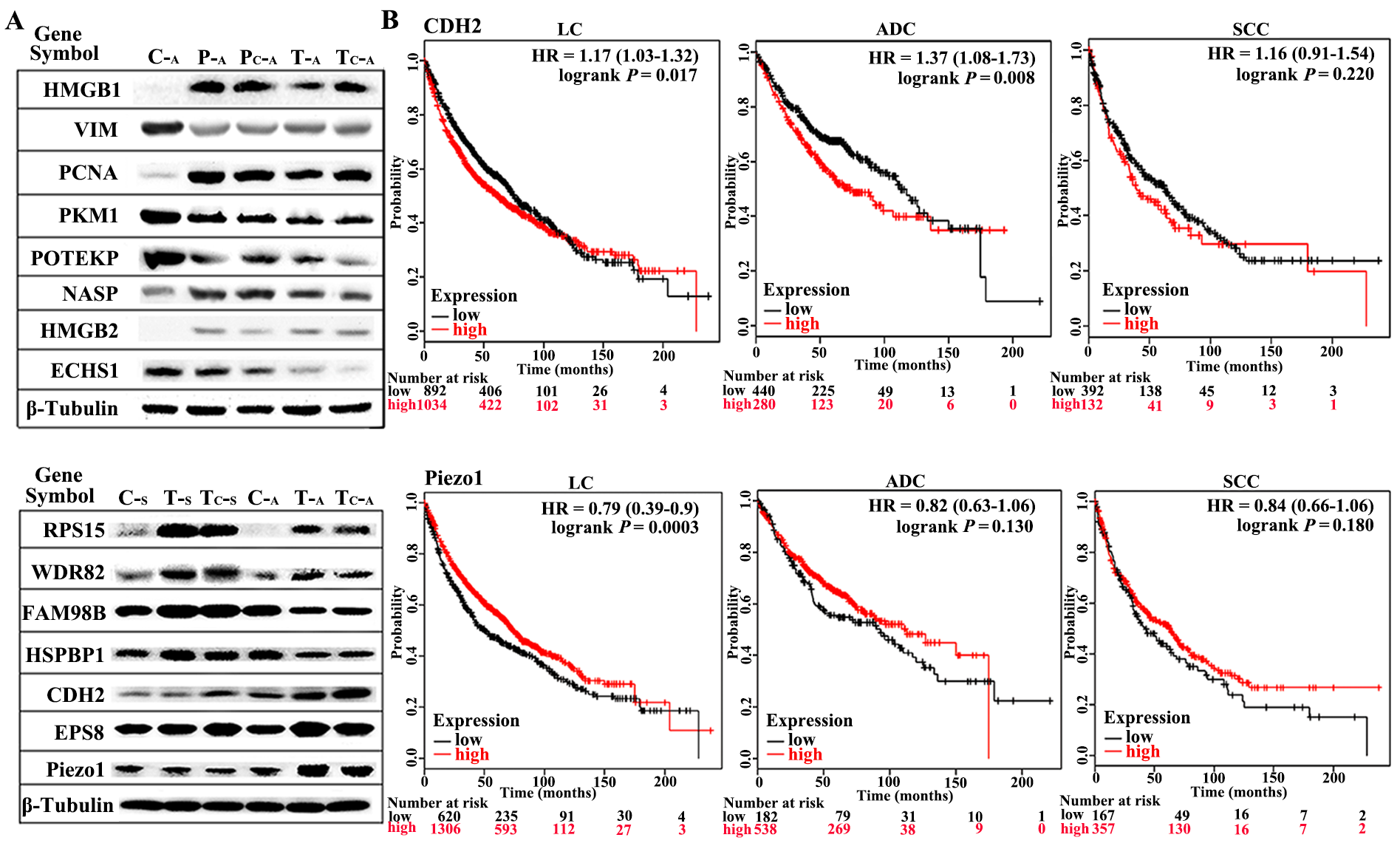

Supplement: Supplementary file 4 — Figure S2. Verification of differential protein expression and the prognostic value of CDH2 and Piezo1 in patients with LC, using the Kaplan–Meier plotter database (http://kmplot.com/analysis/index.php?p=service&cancer= lung). Lysates from C-A (NEC-A), P-A (PEC-A), T-A (TEC-A), Pc-A (PEC-A cocultured with LTEP-α-2), and Tc-A (TEC-A cocultured with LTEP-α-2) cells were prepared from six additional lung ADC patients. Lysates from C-S (NEC-S), T-S (TEC-S), and TC-S (TEC-S cocultured with SK-MES-1) cells were prepared from the six lung SCC patients. The experiments were repeated at least in triplicate. (TIF 6384 kb) [file 12943_2019_987_MOESM4_ESM.tif]

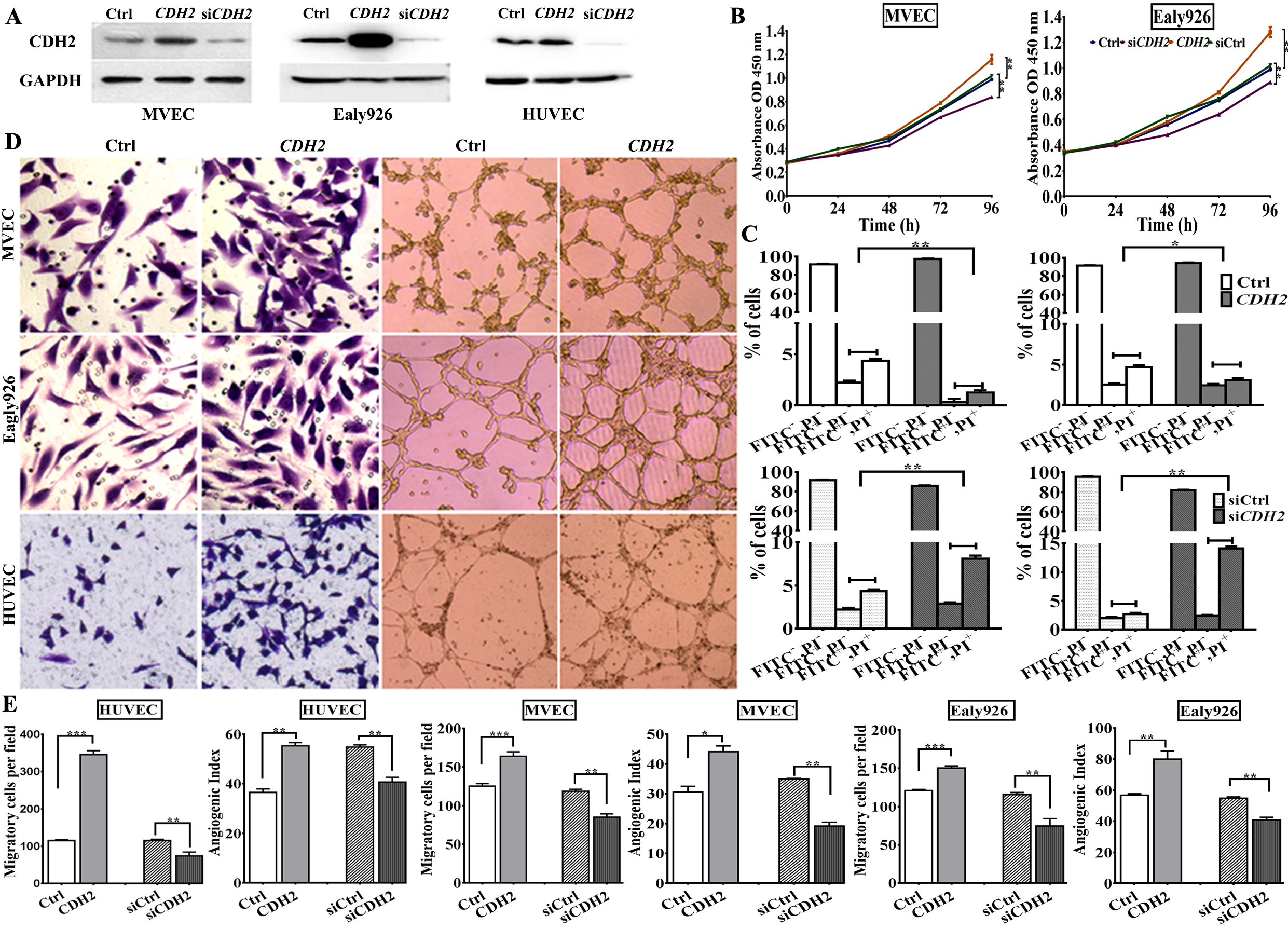

Supplement: Supplementary file 5 — Figure S3. In vitro assays of CDH2 function in microvascular endothelial cell (MVEC), Ealy926, and HUVEC lines by overexpressing or knocking down CDH2 expression. (A) Efficient CDH2 overexpression or knockdown was confirmed by western blotting. (B, C) Cell survival and apoptosis assays of MEVCs and Ealy926 cells after CDH2 overexpression or knock down. (D, E) Cell migration and in vitro angiogenic activity of MVECs, Ealy926 cells and HUVECs following CDH2 overexpression or knock down. Cell numbers were counted in five randomly selected fields under a microscope. (TIF 13045 kb) [file 12943_2019_987_MOESM5_ESM.tif]

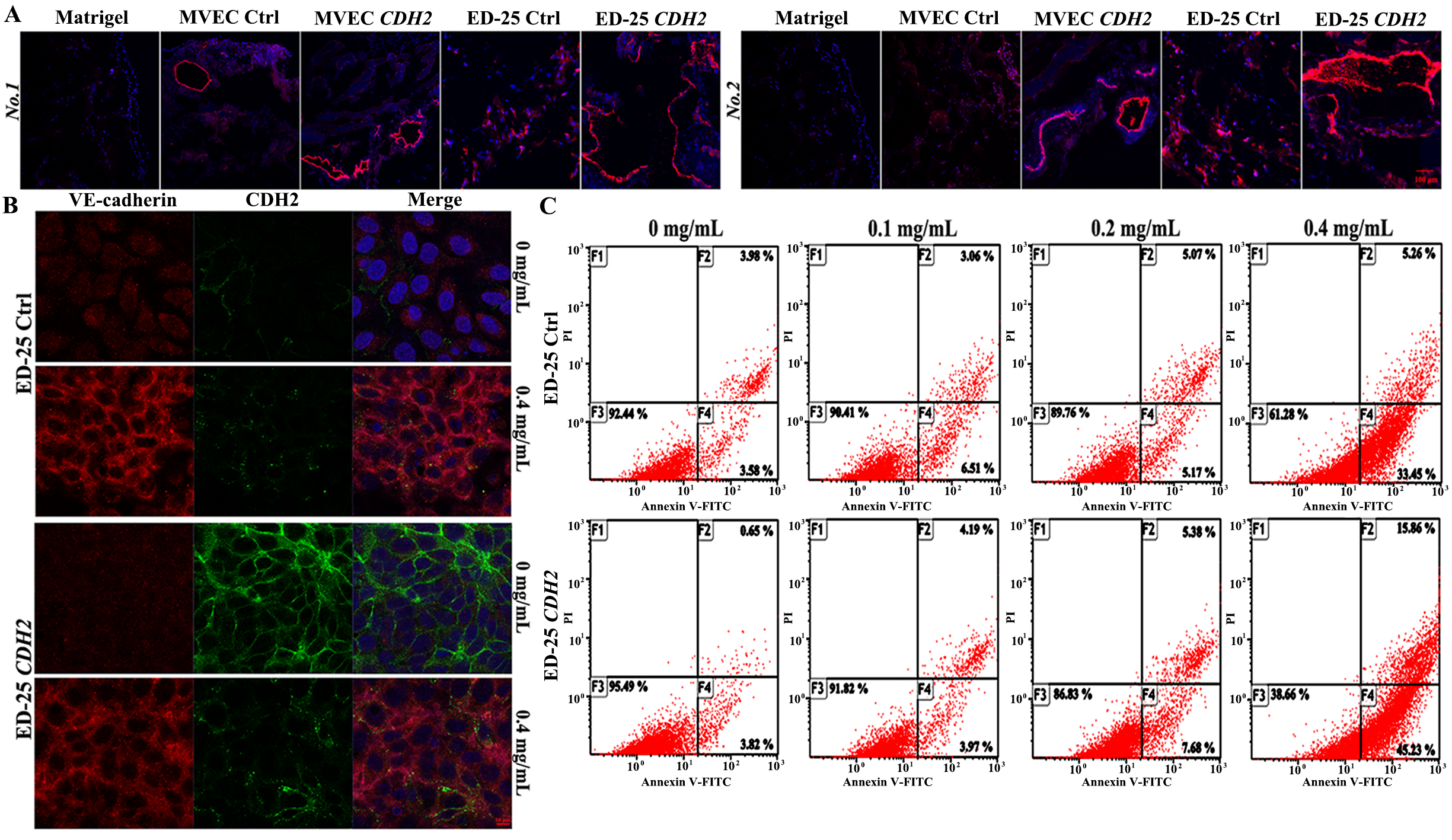

Supplement: Supplementary file 6 — Figure S4. Matrigel plug assay and ADH-1-induced apoptosis in CDH2-expressing cells. (A) Matrigel plug assay. Growth factor-reduced Matrigel matrix (0.25 Ml) supplemented with 8.0 × 10^5^ MVECs or Ealy926 cells (mock cells or clones stably expressing CDH2) was injected subcutaneously at the abdominal midline of six-week-old BALB/c nude male mice. Four mice were included in each group. After 10 days, the Matrigel plugs were split and sectioned for anti-VWF (red) and DAPI (blue) staining. The results from two randomly selected models are shown. (B, C) ADH-1-induced apoptosis in CDH2-expressing cells, as determined by in vitro assays. ADH-1 (0, 0.1, 0.2, or 0.4 mg/mL) was incubated with control and CDH2-overexpressing ED-25 cells for 24 h (confocal microscopy-based observations) or 48 h (FCA). Cells incubated with anti-CDH2 (green) and VE-cadherin antibodies (red) were observed with a confocal microscope. Apoptosis was detected using the Annexin V-FITC/PI Kit. (TIF 10193 kb) [file 12943_2019_987_MOESM6_ESM.tif]

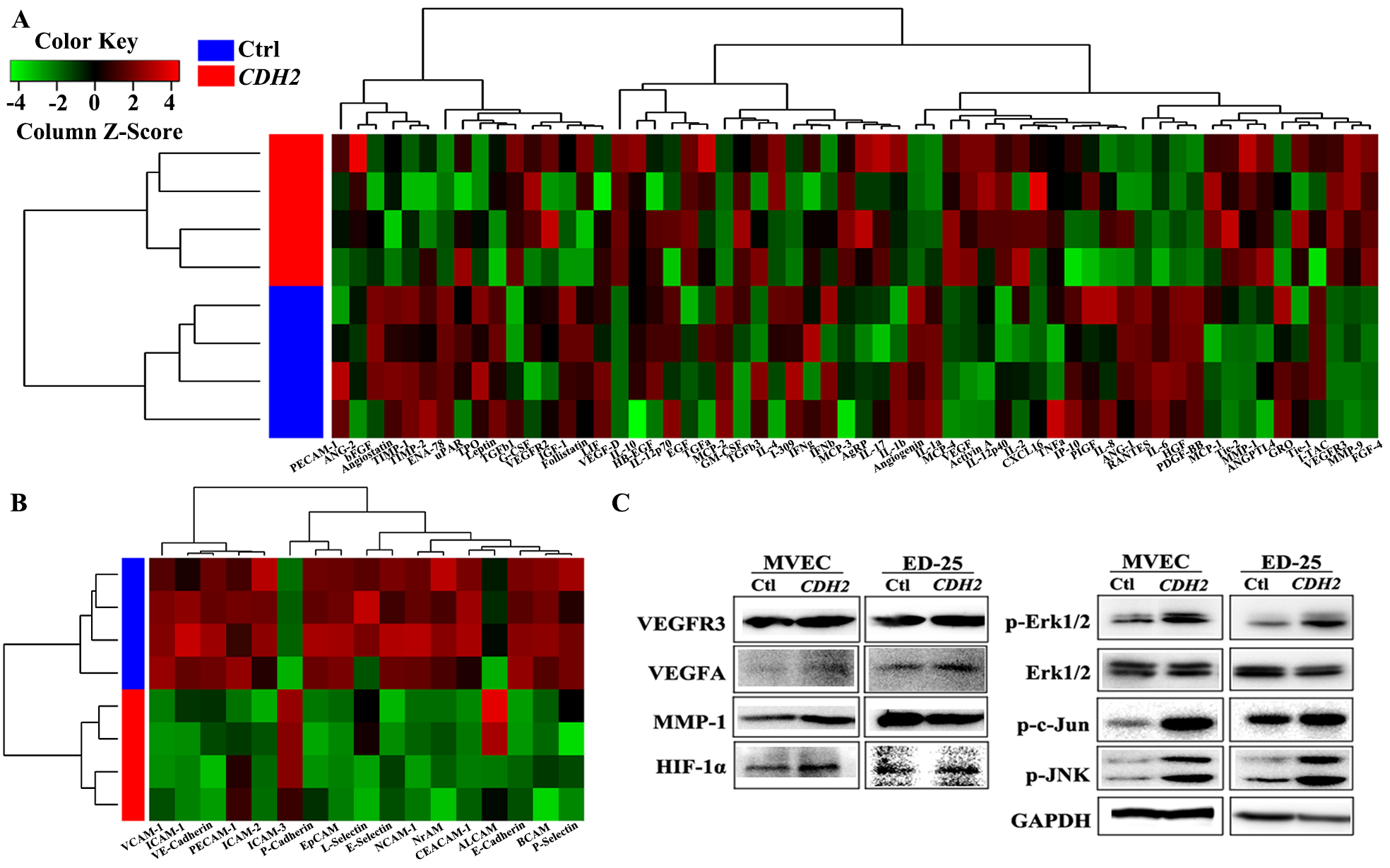

Supplement: Supplementary file 7 — Figure S5. Mechanism of CDH2-mediated angiogenesis. Angiogenesis antibody (A) and molecular adhesion array (B). HUVECs were infected with an adenoviral vector directing the expression of CDH2 or a mock vector. Subsequently, the HUVECs were harvested and analyzed for protein expression using RayBiotech Human Angiogenesis Antibody Array C Series 1000 and Adhesion Molecule Array Q1. The blots were scanned with an InnoScan 300 Microarray Scanner and analyzed using ImageJ software. (C) CDH2 induced upregulation of VEGFA, VEGFR3, MMP-1, and HIF-1α in MVECs and ED-25 cells, as determined by western blotting. Activation of two major MAPK signaling pathways, namely, extracellular signal-regulated kinase (ERK) and c-Jun N-terminal kinase (JNK), was detected. The levels of phosphorylated ERK1/2, JNK, and c-Jun were detected in MVECs and ED-25 cells overexpressing CDH2. (TIF 7116 kb) [file 12943_2019_987_MOESM7_ESM.tif]
