# Supplementary material for: Tumor endothelial cell-derived cadherin-2 promotes angiogenesis and has prognostic significance for lung adenocarcinoma
Source: Mol Cancer. 2019 Mar 4;18:34. doi: 10.1186/s12943-019-0987-1 (PMC6399986; doi:10.1186/s12943-019-0987-1)
Supplement: Supplementary file 3 — Table S1 Proteins differentially expressed in lung adenocarcinoma-derived endothelial cells (TEC-A) in comparison to squamous cell carcinoma-derived endothelial cells (TEC-S). (DOC 68 kb) [file 12943_2019_987_MOESM3_ESM.doc]

**Table S1. Proteins differentially expressed in lung adenocarcinoma-derived endothelial cells (TEC-A) in comparison to squamous cell carcinoma-derived endothelial cells (TEC-S)**

| **No.** | **Accession** | **Gene Symbol** | **Description** | **MW (kDa)** | **PI** | **Coverage (%)** | **# of proteins** | **# of peptides** | **# of unique peptides** | **TEC-A/TEC-SRatio** |
| --- | --- | --- | --- | --- | --- | --- | --- | --- | --- | --- |
| 1 | IPI00167572.4 | FAM98B | Family with sequence similarity 98, member B | 37.17 | 6.29 | 5.76 | 2 | 1 | 1 | 0.27 |
| 2 | IPI00953460.1 | HSPBP1 | HSPA (heat shock 70 kDa) binding protein, cytoplasmic cochaperone | 27.89 | 4.69 | 10.12 | 3 | 1 | 1 | 0.32 |
| 3 | IPI01022358.1 | TUBA1A | Alpha-tubulin | 46.27 | 5.08 | 69.95 | 29 | 2 | 21 | 0.35 |
| 4 | IPI00479058.2 | RPS15 | 40S ribosomal protein S15 | 17.03 | 10.39 | 13.10 | 1 | 1 | 1 | 0.37 |
| 5 | IPI00152695.2 | WDR82 | WD repeat-containing protein 82 | 35.06 | 7.69 | 6.39 | 1 | 1 | 1 | 0.41 |
| 6 | IPI00877866.1 | HP1BP3 | Heterochromatin protein 1, binding protein 3 | 8.23 | 4.84 | 19.74 | 5 | 1 | 1 | 0.42 |
| 7 | IPI00942956.3 | GLUL | Glutamate-ammonia ligase | 26.40 | 6.98 | 3.43 | 2 | 1 | 1 | 0.42 |
| 8 | IPI00910902.1 | NQO1 | NAD(P)H dehydrogenase, quinone 1 | 22.78 | 8.50 | 10.40 | 4 | 1 | 1 | 0.47 |
| 9 | IPI00947340.1 | FKBP8 | FK506 binding protein 8 | 27.53 | 4.59 | 6.32 | 4 | 1 | 1 | 0.51 |
| 10 | IPI00304409.3 | CARHSP1 | Calcium-regulated heat stable protein 1 | 15.88 | 8.21 | 23.81 | 1 | 3 | 3 | 0.54 |
| 11 | IPI01022192.1 | BLOC1S1 | Biogenesis of lysosomal organelles complex-1, subunit 1 | 8.56 | 5.97 | 10.67 | 3 | 1 | 1 | 0.59 |
| 12 | IPI00945920.1 | UMPS | Uridine monophosphate synthetase | 6.15 | 9.52 | 29.82 | 6 | 1 | 1 | 0.59 |
| 13 | IPI00788157.1 | XRN2 | Isoform 2 of 5'-3' exoribonuclease 2 | 99.90 | 8.02 | 2.17 | 3 | 1 | 1 | 0.59 |
| 14 | IPI00410615.2 | ACP1 | Acid phosphatase 1 | 14.33 | 5.58 | 14.52 | 3 | 1 | 1 | 0.60 |
| 15 | IPI00008527.3 | RPLP1 | Ribosomal protein, large, P1 | 11.51 | 4.32 | 14.04 | 1 | 1 | 1 | 0.61 |
| 16 | IPI00081836.3 | HIST1H2AM | Histone H2A type 1-H | 13.90 | 10.89 | 32.81 | 18 | 1 | 4 | 0.62 |
| 17 | IPI00470791.2 | ADI1 | Isoform 2 of 1,2-dihydroxy-3-keto-5-methylthiopentene dioxygenase | 20.32 | 6.29 | 6.94 | 2 | 1 | 1 | 0.65 |
| 18 | IPI00900380.1 | MCTS1 | Isoform 2 of malignant T cell-amplified sequence 1 | 19.22 | 8.25 | 10.06 | 3 | 1 | 1 | 0.66 |
| 19 | IPI00645009.1 | STAU1 | Staufen, RNA binding protein, homolog 1 | 22.46 | 9.95 | 5.53 | 5 | 1 | 1 | 0.67 |
| 20 | IPI00033130.3 | SAE1 | SUMO-activating enzyme subunit 1 | 38.43 | 5.30 | 12.14 | 5 | 2 | 2 | 1.51 |
| 21 | IPI01015923.1 | ITGB1 | Integrin beta 1 | 83.53 | 5.06 | 2.00 | 4 | 1 | 1 | 1.55 |
| 22 | IPI00398779.5 | PLEC | Isoform 4 of plectin | 515.88 | 5.80 | 49.70 | 14 | 1 | 195 | 1.56 |
| 23 | IPI00854764.3 | LMF2 | Isoform 3 of lipase maturation factor 2 | 67.11 | 10.14 | 2.69 | 4 | 1 | 1 | 1.58 |
| 24 | IPI00219426.1 | PVR | Isoform gamma of poliovirus receptor | 39.28 | 6.21 | 3.85 | 3 | 1 | 1 | 1.58 |
| 25 | IPI00976486.1 | TMX4 | Thioredoxin-related transmembrane protein 4 | 12.84 | 3.93 | 13.91 | 2 | 1 | 1 | 1.59 |
| 26 | IPI01021323.1 | NAP1L1 | Nucleosome assembly protein 1-like 1 | 24.35 | 4.51 | 36.23 | 14 | 1 | 5 | 1.63 |
| 27 | IPI01022897.1 | CDK4 | Cyclin-dependent kinase 4 | 12.44 | 7.52 | 11.30 | 8 | 1 | 1 | 1.73 |
| 28 | IPI00743775.1 | CCDC47 | Isoform 2 of coiled-coil domain-containing protein 47 | 55.28 | 4.74 | 5.83 | 2 | 2 | 2 | 1.77 |
| 29 | IPI01009879.1 | EPS8 | Epidermal growth factor receptor pathway substrate 8 | 7.14 | 8.73 | 20.59 | 3 | 1 | 1 | 2.19 |
| 30 | IPI00917650.2 | PIEZO1 | Piezo-type mechanosensitive ion channel component 1 | 286.60 | 7.47 | 0.52 | 1 | 1 | 1 | 2.35 |
| 31 | IPI00658202.1 | CDH2 | Cadherin-2 | 96.98 | 4.84 | 1.71 | 2 | 1 | 1 | 2.41 |
