# Supplementary material for: Tumor endothelial cell-derived cadherin-2 promotes angiogenesis and has prognostic significance for lung adenocarcinoma
Source: Mol Cancer. 2019 Mar 4;18:34. doi: 10.1186/s12943-019-0987-1 (PMC6399986; doi:10.1186/s12943-019-0987-1)
Supplement: Supplementary file 8 — Table S2. Clinical data for 218 lung carcinoma specimens examined in this study. (DOC 274 kb) [file 12943_2019_987_MOESM8_ESM.doc]

**Table S2. Clinical data of 218 lung carcinoma specimens examined in this study**

| NO. | Gender | Age (years) | Type I | Histology grade | Stage | T Stage | Follow-up time (m) | Status |
| --- | --- | --- | --- | --- | --- | --- | --- | --- |
| 1 | M | 61 | S | M | III | T2 | 19 | 1 |
| 2 | F | 59 | S | L | II | T2 |  |  |
| 3 | M | 74 | A | L | I | T2 |  |  |
| 4 | M | 48 | S | M | III | T2 | 35 | 1 |
| 5 | F | 62 | A | M | III | T4 |  |  |
| 6 | M | 44 | A | M | IV | T2 |  |  |
| 7 | F | 49 | A | M | I | T1 | 8 | 1 |
| 8 | M | 76 | A | M | I | T1 |  |  |
| 9 | M | 69 | S | L | II | T2 |  |  |
| 10 | M | 59 | A | M | III | T2 | 67 | 0 |
| 11 | M | 52 | S | M | III | T2 |  |  |
| 12 | M | 47 | A | M | III | T2 | 50 | 1 |
| 13 | F | 61 | A | M | I | T2 |  |  |
| 14 | M | 62 | S | M | II | T3 |  |  |
| 15 | M | 56 | S | M | III | T2 | 24 | 1 |
| 16 | M | 59 | S | M | III | T4 |  |  |
| 17 | M | 54 | S | H | III | T1 |  |  |
| 18 | M | 59 | A | M | I | T2 |  |  |
| 19 | M | 66 | S | M | I | T1 |  |  |
| 20 | M | 68 | S | M | II | T3 |  |  |
| 21 | M | 65 | A | L | II | T2 |  |  |
| 22 | F | 72 | A | L | III | T4 |  |  |
| 23 | M | 54 | A | M | II | T1 |  |  |
| 24 | F | 57 | S | M | II | T3 | 23 | 1 |
| 25 | M | 52 | A | M | III | T1 |  |  |
| 26 | M | 58 | S | M | III | T2 | 18 | 1 |
| 27 | M | 59 | A | L | III | T2 |  |  |
| 28 | F | 55 | A | M | III | T1 |  |  |
| 29 | M | 59 | A | L | I | T1 |  |  |
| 30 | M | 73 | A | L | I | T2 |  |  |
| 31 | M | 59 | A | L | I | T2 |  |  |
| 32 | M | 60 | A | M | I | T1 |  |  |
| 33 | F | 61 | A | L | I | T2 |  |  |
| 34 | F | 59 | A | L | III | T2 |  |  |
| 35 | M | 58 | A |  | II | T2 |  |  |
| 36 | M | 62 | S | M | III | T2 |  |  |
| 37 | M | 59 | S | M | III | T2 |  |  |
| 38 | M | 58 | S | M | II | T2 |  |  |
| 39 | M | 68 | A | M | II | T3 |  |  |
| 40 | M | 70 | S | M | II | T3 |  |  |
| 41 | M | 73 | A | L | III | T4 | 34 | 1 |
| 42 | M | 69 | A | L | III | T2 | 18 | 1 |
| 43 | M | 55 | A | M | II | T2 |  |  |
| 44 | F | 56 | A | M | I | T2 |  |  |
| 45 | M | 46 | S | M | III | T3 |  |  |
| 46 | M | 46 | A | L | II | T2 |  |  |
| 47 | M | 63 | S | M | II | T3 |  |  |
| 48 | M | 66 | A | L | I | T1 |  |  |
| 49 | M | 56 | A | M | III | T2 |  |  |
| 50 | M | 74 | A | M | III | T4 | 31 | 1 |
| 51 | M | 65 | S | M | I | T2 | 27 | 1 |
| 52 | M | 55 | S | M | I | T2 | 67 | 0 |
| 53 | M | 54 | A | L | I | T2 | 67 | 0 |
| 54 | F | 52 | A | M | I | T2 |  |  |
| 55 | F | 59 | A | M | IV | T4 | 39 | 1 |
| 56 | F | 51 | A | M | I | T2 | 67 | 0 |
| 57 | M | 59 | A | L | III | T3 |  |  |
| 58 | M | 56 | A | L | I | T2 | 67 | 0 |
| 59 | F | 59 | A | L | IV | T3 | 9 | 1 |
| 60 | M | 55 | S | L | I | T2 | 66 | 0 |
| 61 | F | 55 | A | H | II | T2 | 56 | 0 |
| 62 | M | 69 | A | M | I | T1 | 66 | 0 |
| 63 | F | 75 | A | L | I | T2 | 66 | 0 |
| 64 | F | 60 | A | M | III | T2 | 46 | 0 |
| 65 | M | 63 | S | L | I | T2 | 46 | 0 |
| 66 | M | 43 | S | L | I | T2 | 66 | 0 |
| 67 | M | 87 | S | M | I | T2 | 36 | 0 |
| 68 | F | 51 | A | L | III | T2 | 54 | 0 |
| 69 | F | 64 | S | M | I | T2 | 9 | 1 |
| 70 | M | 46 | S | L | III | T1 |  |  |
| 71 | M | 67 | A | M | I | T2 |  |  |
| 72 | F | 45 | A | M | IV | T2 | 11 | 1 |
| 73 | M | 68 | A | M | III | T4 | 30 | 1 |
| 74 | F | 65 | A | L | I | T1 |  |  |
| 75 | M | 53 | A | L | I | T2 | 66 | 0 |
| 76 | M | 60 | A | M | II | T2 |  |  |
| 77 | M | 72 | A | L | II | T3 | 47 | 0 |
| 78 | M | 46 | S | L | I | T2 | 65 | 0 |
| 79 | M | 50 | A | M | I | T2 | 45 | 0 |
| 80 | M | 57 | A | L | I | T2 | 47 | 0 |
| 81 | F | 55 | A | L | II | T2 | 40 | 1 |
| 82 | M | 57 | S | M | I | T2 | 45 | 0 |
| 83 | M | 49 | S | M | I | T2 | 45 | 0 |
| 84 | F | 42 | S | L | II | T3 |  |  |
| 85 | F | 45 | A | L | IV | T3 | 31 | 1 |
| 86 | M | 42 | S | M | III | T2 | 17 | 1 |
| 87 | M | 45 | S | L | III | T3 | 46 | 0 |
| 88 | M | 52 | S | M | III | T3 | 64 | 0 |
| 89 | M | 54 | A | L | III | T2 |  |  |
| 90 | M | 69 | A | M | II | T2 |  |  |
| 91 | M | 69 | S | L | II | T2 |  |  |
| 92 | M | 51 | S | L | II | T2 | 29 | 1 |
| 93 | M | 64 | S | M | I | T1 | 64 | 0 |
| 94 | M | 66 | A | M | III | T2 | 7 | 1 |
| 95 | M | 67 | A | H | I | T2 |  |  |
| 96 | M | 72 | A | M | I | T2 | 44 | 0 |
| 97 | M | 80 | A | M | I | T2 | 35 | 1 |
| 98 | F | 47 | S | L | I | T2 | 64 | 0 |
| 99 | M | 77 | A | L | I | T2 | 44 | 0 |
| 100 | F | 55 | A | L | I | T1 | 44 | 0 |
| 101 | M | 65 | S | L | I | T2 | 44 | 0 |
| 102 | M | 47 | S | M | III | T3 | 34 | 1 |
| 103 | M | 55 | A | L | IV | T2 | 53 | 0 |
| 104 | M | 56 | A | H | III | T2 |  |  |
| 105 | F | 57 | A | M | III | T2 | 14 | 1 |
| 106 | M | 68 | A | H | II | T1 | 63 | 0 |
| 107 | M | 58 | A | M | I | T1 | 43 | 0 |
| 108 | M | 73 | A | M | I | T2 | 39 | 1 |
| 109 | F | 38 | S | H | II | T3 |  |  |
| 110 | M | 72 | S | H | II | T2 | 63 | 0 |
| 111 | M | 39 | A | M | I | T2 |  |  |
| 112 | M | 61 | A | L | I | T2 | 63 | 0 |
| 113 | M | 74 | S | M | II | T1 |  |  |
| 114 | M | 56 | A | L | II | T2 | 45 | 0 |
| 115 | F | 72 | S | M | II | T3 | 8 | 1 |
| 116 | F | 56 | A | M | III | T2 |  |  |
| 117 | M | 75 | A | L | I | T2 | 43 | 0 |
| 118 | F | 48 | A | L | III | T4 | 9 | 1 |
| 119 | M | 60 | A | M | I | T2 | 43 | 0 |
| 120 | F | 61 | A | H | III | T2 | 12 | 1 |
| 121 | F | 32 | A | M | II | T2 |  |  |
| 122 | M | 51 | A | M | III | T4 |  |  |
| 123 | M | 61 | S | L | I | T2 | 62 | 0 |
| 124 | F | 57 | A | M | III | T2 |  |  |
| 125 | M | 51 | S | H | I | T1 | 62 | 0 |
| 126 | M | 51 | S | L | IV | T2 |  |  |
| 127 | M | 32 | A | L | III | T2 | 21 | 1 |
| 128 | F | 55 | A | L | II | T2 |  |  |
| 129 | F | 57 | A | M | III | T4 |  |  |
| 130 | M | 62 | A | M | I | T2 | 62 | 0 |
| 131 | F | 53 | A | M | I | T2 | 62 | 0 |
| 132 | M | 52 | S | L | II | T2 | 62 | 0 |
| 133 | F | 72 | A | M | I | T1 | 62 | 0 |
| 134 | M | 72 | S | L | II | T3 | 8 | 1 |
| 135 | F | 60 | A | M | III | T2 |  |  |
| 136 | F | 50 | A | L | III | T4 |  |  |
| 137 | M | 61 | S | M | I | T1 | 62 | 0 |
| 138 | F | 70 | A | M | III | T4 | 36 | 1 |
| 139 | M | 75 | A | H | I | T2 | 50 | 0 |
| 140 | F | 62 | A | H | I | T1 | 35 | 1 |
| 141 | F | 65 | A | M | I | T2 | 61 | 0 |
| 142 | M | 45 | A | M | III | T2 | 61 | 0 |
| 143 | M | 61 | A | M | II | T2 | 49 | 0 |
| 144 | M | 59 | A | L | I | T2 | 61 | 0 |
| 145 | M | 51 | A | L | I | T2 | 32 | 1 |
| 146 | M | 59 | A | M | I | T2 | 41 | 0 |
| 147 | F | 65 | A | M | I | T1 | 61 | 0 |
| 148 | F | 53 | A | M | III | T1 |  |  |
| 149 | M | 47 | A | L | III | T2 | 17 | 1 |
| 150 | F | 62 | A | L | III | T4 | 31 | 1 |
| 151 | M | 67 | A | M | III | T2 |  |  |
| 152 | M | 55 | S | M | I | T2 | 61 | 0 |
| 153 | M | 40 | A | L | IV | T2 | 30 | 1 |
| 154 | M | 61 | S | M | I | T3 | 62 | 0 |
| 155 | M | 60 | A | M | IV | T3 | 42 | 0 |
| 156 | M | 67 | A | L | I | T1 | 61 | 0 |
| 157 | M | 64 | S | L | I | T2 | 62 | 0 |
| 158 | M | 52 | A | L | III | T2 | 7 | 1 |
| 159 | M | 38 | S | M | I | T2 | 62 | 0 |
| 160 | M | 72 | S | L | I | T2 | 62 | 0 |
| 161 | F | 77 | A | M | I | T1 | 41 | 0 |
| 162 | M | 54 | S | L | II | T4 |  |  |
| 163 | F | 74 | A | L | III | T3 | 24 | 1 |
| 164 | M | 55 | A | L | III | T4 |  |  |
| 165 | F | 53 | A | M | II | T2 | 61 | 0 |
| 166 | M | 69 | S | L | I | T2 | 47 | 0 |
| 167 | M | 58 | S | M | IV | T4 | 18 | 1 |
| 168 | M | 35 | S | M | I | T2 | 61 | 0 |
| 169 | M | 56 | S | L | III | T4 | 61 | 0 |
| 170 | F | 68 | A | L | III | T2 | 28 | 1 |
| 171 | F | 62 | S | L | III | T3 | 17 | 1 |
| 172 | M | 67 | A | M | IV | T4 |  |  |
| 173 | F | 57 | A | L | I | T2 | 61 | 0 |
| 174 | M | 63 | A | M | III | T2 | 41 | 1 |
| 175 | M | 66 | A | L | III | T2 | 39 | 1 |
| 176 | M | 63 | S | L | I | T2 | 60 | 0 |
| 177 | M | 57 | A | M | III | T2 | 61 | 0 |
| 178 | M | 73 | A | L | I | T2 | 42 | 0 |
| 179 | M | 62 | A | M | III | T2 | 18 | 1 |
| 180 | M | 66 | S | M | III | T3 | 49 | 1 |
| 181 | M | 60 | S | M | I | T2 | 61 | 0 |
| 182 | M | 70 | A | M | I | T2 | 61 | 0 |
| 183 | M | 65 | S | L | II | T1 | 44 | 1 |
| 184 | F | 40 | A | L | III | T3 | 61 | 0 |
| 185 | M | 55 | S | M | III | T2 |  |  |
| 186 | M | 47 | A | L | III | T2 |  |  |
| 187 | M | 54 | A | L | I | T2 |  |  |
| 188 | M | 59 | S | M | I | T1 | 59 | 0 |
| 189 | M | 58 | S | M | III | T2 | 58 | 0 |
| 190 | M | 51 | A | M | III | T2 | 58 | 0 |
| 191 | F | 63 | A | M | I | T1 |  |  |
| 192 | M | 51 | A | L | IV | T4 | 58 | 0 |
| 193 | M | 55 | A | L | III | T2 | 18 | 1 |
| 194 | M | 56 | A | L | III | T1 |  |  |
| 195 | M | 59 | S | M | II | T2 |  |  |
| 196 | F | 57 | A | M | III | T2 | 58 | 0 |
| 197 | M | 34 | A | L | III | T2 |  |  |
| 198 | F | 54 | A | M | II | T2 | 58 | 0 |
| 199 | F | 39 | S | L | I | T2 | 58 | 0 |
| 200 | F | 35 | A | M | III | T2 | 58 | 0 |
| 201 | M | 40 | A | M | III | T2 | 58 | 0 |
| 202 | F | 64 | A | M | I | T2 |  |  |
| 203 | M | 61 | S | L | I | T2 | 35 | 1 |
| 204 | M | 61 | S | M | I | T2 | 58 | 0 |
| 205 | M | 58 | S | M | III | T3 | 57 | 0 |
| 206 | F | 58 | A | M | I | T2 | 39 | 0 |
| 207 | M | 53 | A | M | III | T4 | 19 | 1 |
| 208 | F | 70 | A | L | II | T2 | 46 | 0 |
| 209 | M | 42 | S | L | III | T2 | 57 | 0 |
| 210 | F | 46 | A | L | I | T2 | 39 | 0 |
| 211 | F | 60 | A | M | I | T2 |  |  |
| 212 | F | 62 | A | M | I | T1 | 39 | 0 |
| 213 | F | 65 | A | M | I | T2 | 33 | 1 |
| 214 | F | 71 | A | L | III | T1 | 43 | 0 |
| 215 | M | 66 | S | L | I | T2 |  |  |
| 216 | M | 55 | A | M | I | T2 |  |  |
| 217 | F | 54 | S | M | I | T2 | 47 | 0 |
| 218 | M | 73 | S | L | III | T2 | 36 | 1 |

Notes: Gender: female (F), male (M); Type I: squamous cell carcinoma (S), adenocarcinoma (A); histological grade: high differentiation(H), moderate differentiation(M), low differentiation (L); status: death (1), censored data (0)
