# Supplementary material for: Tumor endothelial cell-derived cadherin-2 promotes angiogenesis and has prognostic significance for lung adenocarcinoma
Source: Mol Cancer. 2019 Mar 4;18:34. doi: 10.1186/s12943-019-0987-1 (PMC6399986; doi:10.1186/s12943-019-0987-1)
Supplement: Supplementary file 9 — Table S3. Expression of CDH2 in tumor-derived endothelial cell and its correlation with clinicopathological features of patients with NSCLC. (DOC 82 kb) [file 12943_2019_987_MOESM9_ESM.doc]

**Table S3. Expression of CDH2 in tumor-derived endothelial cell and its correlation with clinicopathological features of patients with NSCLC**

| Patients | Number | CDH2—LC | | | | CDH2—ADC | CDH2—SCC | | Type 1 | |
| --- | --- | --- | --- | --- | --- | --- | --- | --- | --- | --- |
|  |  | Score | *P-*value (Person correlation) | | *P-*value (Partial correlation) | *P-*value (Person correlation) | *P-*value (Person correlation) |  | | *P-*value (Person correlation) |
| Gender | | | | |  |  |  |  | |  |
| Female | 67 | 3.82 ± 0.20 | | 0.012**^＊^** | 0.329 | 0.843 | 0.104 |  | | 0.000**^＊＊＊^** |
| Male | 151 | 3.20 ± 0.14 | |  |  |  |  |  | |  |
| Age (years) | | | | |  |  |  |  | |  |
| ≤ Average (59) | 105 |  | | 0.362 | 0.639 | 0.588 | 0.044**^＊^** |  | | 0.477 |
| > Average | 113 |  | |  |  |  |  |  | |  |
| Smoke history | | | | |  |  |  |  | |  |
| No | 64 | 4.93 ± 0.39 | | 0.011**^＊^** | 0.664 | 0.695 | 0.678 |  | | 0.004**^＊＊^** |
| Yes | 154 | 3.56 ± 0.29 | |  |  |  |  |  | |  |
| Histology grade | | | | |  |  |  |  | |  |
| Well differentiated | 12 |  | | 0.339 | 0.892 | 0.126 | 0.772 |  | | 0.879 |
| Moderately differentiated | 117 |  | |  |  |  |  |  | |  |
| Poorly differentiated | 89 |  | |  |  |  |  |  | |  |
| Stage | | | | |  |  |  |  | |  |
| I | 90 | 3.10 ± 0.17 | | 0.042**^＊^** | 0.038**^＊^** | 0.044**^＊^** | 0.522 |  | | 0.389 |
| II | 39 | 3.45 ± 0.30 | |  |  |  |  |  | |  |
| III–IV | 89 | 3.65 ± 0.18 | |  |  |  |  |  | |  |
| Lymph node metastasis | | | | |  |  |  |  | |  |
| Absent | 167 |  | | 0.855 | 0.217 | 0.468 | 0.681 |  | | 0.236 |
| Present | 51 |  | |  |  |  |  |  | |  |
| Tumor size | | | | |  |  |  |  | |  |
| Size ≤ 3 cm | 116 | 4.33 ± 0.36 | | 0.011**^＊^** | 0.909 | 0.768 | 0.900 |  | | 0.000**^＊＊＊^** |
| 3 < size ≤ 5 cm | 22 | 4.57 ± 0.50 | |  |  |  |  |  | |  |
| 5 < size ≤ 7 cm | 36 | 3.18 ± 0.46 | |  |  |  |  |  | |  |
| Size > 7 cm | 44 | 2.80 ± 0.66 | |  |  |  |  |  | |  |
| T stage | | | | |  |  |  |  | |  |
| T1 | 32 |  | | 0.607 | 0.964 | 0.370 | 0.790 |  | | 0.181 |
| T2 | 141 |  | |  |  |  |  |  | |  |
| T3 | 25 |  | |  |  |  |  |  | |  |
| T4 | 20 |  | |  |  |  |  |  | |  |
| Type 1 | | | | |  |  |  |  | |  |
| Squamous cell carcinoma | 77 | 2.35 ± 0.14 | | 0.000**^＊＊＊^** | 0.000**^＊＊＊^** | - | - |  | | - |
| Adenocarcinoma | 141 | 3.93 ± 0.14 | |  |  |  |  |  | |  |
| Type 2 | | | | |  |  |  |  | |  |
| Central | 10 | 2.78 ± 0.29 | | 0.000**^＊＊＊^** | 0.371 | 0.360 | 0.921 |  | | 0.000**^＊＊＊^** |
| Peripheral | 208 | 4.94 ± 0.30 | |  |  |  |  |  | |  |
| Visceral pleura metastasis | | | | |  |  |  |  | |  |
| Absent | 101 |  | | 0.242 | 0.224 | 0.037**^＊^** | 0.727 |  | | 0.029**^＊^** |
| Present | 117 |  | |  |  |  |  |  | |  |
| Macrovascular invasion | | | | |  |  |  |  | |  |
| Absent | 152 |  | | 0.686 | 0.132 | 0.365 | 0.820 |  | | 0.185 |
| Present | 66 |  | |  |  |  |  |  | |  |
| Neural invasion | | | | |  |  |  |  | |  |
| Absent | 130 | 4.17 ± 0.23 | | 0.020**^＊^** | 0.555 | 0.453 | 0.816 |  | | 0.001**^＊＊^** |
| Present | 88 | 3.54 ± 0.25 | |  |  |  |  |  | |  |

Notes: CDH2—LC: expression of CDH2 in TECs of all LC patients; CDH2—ADC: expression of CDH2 in TECs of ADC samples; CDH2—SCC: expression of CDH2 in TECs of SCC samples; Type 1: ADC and SCC. Clinical data were analyzed with Pearson and Partial correlation coefficient, which is used to exclude the effects of control variable. *P* < 0.05**^＊^**; *P* < 0.01**^＊＊^**; *P* < 0.001**^＊＊＊^** (2-tailed).
